# Psychological effects of advanced care on patients received endoscopic gastric cancer resection

**DOI:** 10.1097/MD.0000000000017497

**Published:** 2019-10-11

**Authors:** Xiao-li Cao, Xin Wang, Ping Li, Wei Ju

**Affiliations:** Department of Gastroenterology, Xi’an No.9 Hospital, Xi’an, China.

**Keywords:** advance care, anxiety, depression, gastric cancer

## Abstract

**Background:**

This study will systematically evaluate the psychological effects of advanced care (AC) on patients who received endoscopic gastric cancer resection (EGCR).

**Methods:**

This study will search the following databases of Cochrane Library, Pubmed, EMBASE, Web of Science, WANGFANG, Chinese Biomedical Literature Database, and China National Knowledge Infrastructure from inception to the present with no language limitation. All randomized controlled trials on assessing the psychological effects of AC for patients with EGCR will be included.

**Results:**

This study will explore the psychological effects of AC on EGCR by assessing depression, anxiety, health-related quality of life, and adverse events.

**Conclusion:**

This study will summarize recent evidence for the psychological effects of AC on EGCR.

**PROSPERO registration number:**

PROSPERO CRD42019139868.

## 1 Introduction

Gastric cancer (GC) is one of the most common cancers around the world.^[1–3]^ It is often characterized as indigestion and stomach discomfort, a bloated feeling after eating, mild nausea, loss of appetite, and heartburn at the early stage.^[4–6]^ At the advanced stage, it manifests as blood in the stool, vomiting, weight loss for no known reason, stomach pain, jaundice, ascites, and trouble swallowing.^[7–9]^ It has been reported that >951,000 people have been diagnosed as GC annually as new cases, and about 723,000 died from such disease.^[10,11]^ Surgical resection of GC, especially for endoscopic gastric cancer resection (EGCR), is widely used to manage GC.^[12–18]^ Previous studies found that patients with GC who received EGCR often have psychological disorders.^[19–32]^ Although numerous studies reported that advanced care (AC) can help relieve psychological disorders in GC patients who received EGCR.^[20–32]^ However, no evidence on levels of evidence-based medicine is provided. Therefore, in this study, we will systematically assess the psychological effects of AC on patients who underwent EGCR.

## 2 Methods

### 2.1 Criteria for considering studies for this study

#### 2.1.1 Study types

We will consider randomized controlled trials (RCTs) that assess the psychological effects of AC in patients received EGCR. All non-RCTs, such as animal studies, case studies, observational studies will be excluded.

#### 2.1.2 Participant types

People of any age, in any setting who are diagnosed as EGCR, will be included in this study.

#### 2.1.3 Intervention types

Patients in the experimental group underwent AC in this study.

Patients in the control group received any interventions except AC in this study.

#### 2.1.4 Outcome types

The primary outcome is depression. It can be measured by any associated scales, such as Hamilton Depression Rating Scale.

The secondary outcomes consist of anxiety and health-related quality of life. Anxiety is assessed via Hamilton Anxiety Rating Scale or other tools. Health-related quality of life is evaluated by any relevant scales, including 36-Item Short Form Health Survey. In addition, adverse events will also be assessed.

### 2.2 Search methods

We will search the databases of Pubmed, Cochrane Library, EMBASE, Web of Science, WANGFANG, Chinese Biomedical Literature Database, and China National Knowledge Infrastructure from inception to the present with no language limitation. In addition, gray literature will also be searched, such as conference proceedings, dissertations, and reference lists of associated reviews. The detailed search strategy for Cochrane Library is showed in Table 1. We will utilize the similar search strategy to other electronic databases.

**Table 1:**
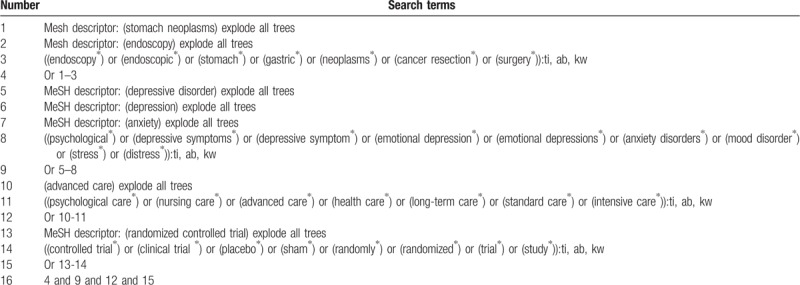
Search strategy utilized for Cochrane Library.

### 2.3 Data collection

#### 2.3.1 Study selection

Two authors will independently screen all titles and abstracts retrieved to check whether they meet inclusion criteria. We will obtain full papers of the remaining papers for judging their eligibility criteria. Any divergences between 2 authors will be solved with a third author to make a consensus decision by discussion. The results of study selection will be showed in a flowchart of based on the guideline of Preferred Reporting Items for Systematic Reviews and Meta-Analyses.

#### 2.3.2 Data extraction

Two authors will independently extract data from all eligible studies using a data extraction form. Any disagreements will be solved by another author through discussion. Specifically, the following information will be extracted:

Title, primary author, year of publication, location
Study setting
Eligibility criteria, diagnostic criteria
Patient characteristics
Sample size
Study quality, such as randomization, allocation concealment, blinding, among others
Intervention details, comparators
Primary and secondary outcomes, safety
Follow-up visit information
Funding source.

#### 2.3.3 Missing data management

If essential data are missing or insufficient, we will obtain them from primary authors using email. We will analyze available data if we can not obtain these data.

### 2.4 Risk of bias evaluation

Independently, 2 authors will evaluate the risk of bias of eligible studies using Cochrane Risk of Bias Tool. We will invite a third author to solve any divisions between 2 authors. In addition, we will exert our evaluation of risk of bias using “Risk of bias" summary figures.

### 2.5 Measures of treatment effect

For continuous outcome values, mean difference or standardized mean difference will be calculated with 95% confidence intervals. For dichotomous outcome values, risk ratio will be calculated with 95% confidence intervals.

### 2.6 Assessment of heterogeneity

Clinical heterogeneity will be evaluated using *I*^2^ test. Where *I*^2^ values are ≤50%, heterogeneity is considered as low, and a fixed-effects model will be used. Where *I*^2^ values exceed 50%, heterogeneity is regarded as significant, and a random-effects model will be utilized.

### 2.7 Data synthesis and analysis

RevMan 5.3 software is utilized to perform statistical analysis. We will pool the data where the *I*^2^ values are ≤50%, and meta-analysis will be carried out. We will not pool the data where the *I*^2^ values are >50%, and subgroup analysis will be performed. We will report narrative summary and will not include any meta-analysis if there is still significant heterogeneity after subgroup analysis.

### 2.8 Additional analysis

If substantial heterogeneity exists among eligible studies, we will conduct subgroup analysis to investigate reasons for heterogeneity based on the different study quality, treatments, and outcome measurements.

Additionally, sensitivity analysis will be carried out to identify the robustness of pooled outcome results by removing high risk of bias studies.

### 2.9 Reporting bias

We will perform Funnel plot^[33]^ and Egger regression test^[34]^ to check reporting bias when sufficient studies are included.

### 2.10 Ethics and dissemination

No ethical approval is inquired because this study will only analyze previously published data. We will publish this study at a peer-reviewed journal.

## 3 Discussion

GC is one of the most common cancers globally, which leads to high mortality.^[1,2]^ Although EGCR is often widely utilized to treat such disorder effectively, most patients experience psychological disorders.^[19–32]^ Previous studies have reported that AC can benefit GC patients who received EGCR. However, its results are still inconsistent on the levels of evidence-based medicine. Therefore, this study will assess the psychological effects of AC on the GC patients who undergo EGCR. The results of this study may summarize the evidence of AC for psychological outcomes in GC patients received EGCR across all published RCTs.

## Author contributions

**Conceptualization:** Xiao-li Cao, Xin Wang, Wei Ju.

**Data curation:** Xiao-li Cao, Ping Li.

**Formal analysis:** Xiao-li Cao, Xin Wang, Ping Li.

**Investigation:** Xiao-li Cao.

**Methodology:** Xin Wang, Ping Li, Wei Ju.

**Project administration:** Xiao-li Cao.

**Resources:** Xin Wang, Ping Li, Wei Ju.

**Software:** Xin Wang, Ping Li, Wei Ju.

**Supervision:** Xiao-li Cao.

**Validation:** Xiao-li Cao, Xin Wang, Wei Ju.

**Visualization:** Xiao-li Cao, Ping Li.

**Writing – original draft:** Xiao-li Cao, Xin Wang, Ping Li, Wei Ju.

**Writing – review & editing:** Xiao-li Cao, Xin Wang, Ping Li, Wei Ju.
